# Overfertilization reduces tomato yield under long-term continuous cropping system *via* regulation of soil microbial community composition

**DOI:** 10.3389/fmicb.2022.952021

**Published:** 2022-08-04

**Authors:** Qiaobo Song, Hongdan Fu, Qingwen Shi, Xuan Shan, Zhen Wang, Zhouping Sun, Tianlai Li

**Affiliations:** ^1^National & Local Joint Engineering Research Center of Northern Horticultural Facilities Design & Application Technology, Shenyang, China; ^2^Key Laboratory of Protected Horticulture of Education Ministry and Liaoning Province, College of Horticulture, Shenyang Agriculture University, Shenyang, China

**Keywords:** long-term continuous cropping, overfertilization, soil acidification, microbial community, structural equation model

## Abstract

Long-term monoculture cropping and overfertilization degrade soil fertility, which reduces crop growth and promotes the development of soil-borne diseases. However, it remains unclear what the temporal effects of the above factors are on the tomato yield and microbial community structure. Thus, a greenhouse experiment with different amounts of fertilization [2,196 kg ha^−1^ (control) and 6,588 kg ha^−1^ (overfertilization) of inorganic fertilizers (NPK)] was carried out with the soils used previously for 1, 2, and 12 years under monoculture of tomato. A 12-year overfertilization decreased soil pH by 1.37 units. Soil electrical conductivity (EC) and concentrations of soil nutrients are enhanced with the increase in tomato cropping duration. Higher content of soil nutrients was found under overfertilization compared to the control in the 12-year soil. Overfertilization decreased the activity of β-1,4-glucosidase (BG) and oxidase compared to the control in the 12-year soil. Bacterial diversity and richness decreased by 6 and 31%, respectively, under overfertilization in 12-year soil compared to the control. The relative abundance of *Gemmatimonas* and *Gp6* in 12-year soil under overfertilization was 17 and 78%, respectively, lower than in control soil. Soil pH and total carbon (TC) were the major factors explaining changes in microbial composition. A 38% decrease in yield was caused by overfertilization in 12-year soil compared to the control. Microbial community composition was the main factor that moderated tomato yield. In addition, fertilization rather than cropping duration had a greater impact on tomato yield. Therefore, our results suggest that long-term overfertilization influenced soil pH, soil TC, and soil microbial community composition to regulate tomato yield.

## Introduction

Continuous monoculture cropping is a common cultural pattern in the greenhouse, and lots of research has found that the yield and quality of crops are reduced under this system. This occurs due to (i) soil acidification and secondary salinization (Shi et al., 2009; Han et al., 2014) caused by the reduced leaching with rainwater and application of inorganic fertilizer; (ii) production and accumulation of allelochemicals (fatty acids and phenolic acids) (Bertin et al., 2003), which influence the permeability of root membranes, nutrient uptake, enzyme activities, and microbial community development (Ning et al., 2020; Shi et al., 2021) and, thus, affect the growth of crops (Chung et al., 2000; Wu et al., 2007); and (iii) soil-borne diseases such as *Fusarium* wilt, which reduce the quality and yield of vegetables (Reeves, 1997; Yu, 2001; Zhao et al., 2019). Besides, selective nutrient accumulation is observed (Magarey, 1999), which, together with consistent fertilization, further promotes nutrient imbalance (Ning et al., 2020). Long-term continuous cropping decreases soil microbial diversity, which simplifies community structure, the number and types of beneficial bacteria (e.g., *Mortierella* and *Pseudaleuria*), and keystone taxa (e.g., *Acidobacteria Gp1, Ac-idobacteria Gp2*, and *Acidobacteria 16*) abundances, which unbalances the community (Ning et al., 2020; Tan et al., 2021), but not enough information is available about the importance of these impacts on crop growth.

Fertilization can not only increase the content of soil organic matter and stabilize the microbial community and enzyme activity, but it can also improve plants and microorganisms' nutrient use efficiency (Xing and Zhu, 2002; Yang et al., 2018; Puissant et al., 2019). However, excessive inorganic fertilizer input in greenhouse production driven by yield and economic benefit is common, especially for N fertilizer (Guo et al., 2010; Ali et al., 2020). The fertilizer N application rates used for vegetable production in North China ranged from approximately 500 to 1,900 kg N ha^−1^ (Zhang et al., 1996). N fertilizer application rates reached 4,000 kg N ha^−1^ in Shandong province (Ju et al., 2007). In tomato cultivation, higher nitrogen use efficiency, biomass, soil organic C and organic N, and lower N leaching were observed in relatively low N application rate (from 143 to 360 kg N ha^−1^) treatments than in relatively high N application rate (from 480 to 870 kg N ha^−1^) treatments (Sainju et al., 2001; Liang et al., 2020). Long-term low fertilization (120 kg N ha^−1^) does not change the pH and the electrical conductivity (EC; Masto et al., 2007; Xu et al., 2012). However, overfertilization (900 kg N ha^−1^) introduced soil-borne pathogens, such as *Fusarium oxysporum* and *Luteimonas*, and suppressed the beneficial microorganisms, including nitrifiers, and *Nocardioides, Ilumatobacter*, and *Gaiella* (Zhang X. M. et al., 2017; Zhao et al., 2019). Short-term overfertilization causes beneficial effects due to the provision of sufficient nutrients for crops, while with the increase in cropping duration, the beneficial effects gradually decrease or even disappear (Lazcano et al., 2013; Han et al., 2016). Long-term overfertilization decreased the soil pH (Zhang Y. T. et al., 2017); increased the content of NH${}_{4}^{+}$ (Ma et al., 2021); caused a greater risk of N leaching; enhanced nitrous oxide emissions (Song et al., 2016); reduced the activity of β-glucosidase and phosphatases; affected C, N, and P cycling; and reduced the content of base cations (Chang et al., 2007). Furthermore, long-term fertilization reduced bacterial diversity, richness, and fungal diversity (Wallenstein et al., 2006; Yuan et al., 2020); enhanced fungal richness (Dong et al., 2014); and altered the community structural and functional gene structure of microbes in soil (Edwards et al., 2011; Su et al., 2015). Inorganic fertilization decreased the abundance of microbial taxa (i.e., phyla *Proteobacteria, Bacteroidetes, Mortierella, and Pseudaleuria*), which are associated with plant growth, organic matter accumulation, and disease suppression (Ma et al., 2018; Ning et al., 2020). However, there is not enough data on how organic fertilization combined with different concentrations of inorganic fertilization could affect soil properties *via* long-term continuous cropping.

Despite long-term continuous cropping and overfertilization coexist under the greenhouse culture system, there are only a few reports on how these two variables interact and affect the crop yield. Thus, this study used soils from continuous cropping for 1, 2, and 12 years to reveal the effect of usual (2,196 kg ha^−1^) vs. overfertilization (6,588 kg ha^−1^) of inorganic fertilizers combined with the same amount of chicken manure on the yield of tomato (*Solanum lycopersicum* L.), soil chemical properties, and microbial communities. It was hypothesized that (i) control and overfertilization will alter the yield of tomatoes differently under long-term monoculture cropping, and (ii) microbial community activity will differ among control and overfertilization due to alteration of soil properties.

## Materials and methods

### Soil sampling from the field

Two types of long-term greenhouse fertilized soils, which have been treated for 0, 1, and 11 years of continuous tomato monoculture with rational fertilization (control) and overfertilization, were collected from two adjacent sites in Liaoning Province, China. The fertilization strategies of these two soils were as follows: control—chemical fertilizers [ShiJiaLi Chemical fertilizer company limited (Chengdu, China)] were applied at rates of 286 kg N ha^−1^, 229 kg P_2_O_5_ ha^−1^, and 190 kg K_2_O ha^−1^; and overfertilization—chemical fertilizers were applied at rates of 858 kg N ha^−1^, 685 kg P_2_O_5_ ha^−1^, and 571 kg K_2_O ha^−1^. Chicken manure compost [2.80 g N kg^−1^, 2.43 g P_2_O_5_ kg^−1^, and 0.13 g K_2_O kg^−1^; RuiYuanDe Biotechnology Co., Ltd. (Shenyang, China)] was added to both soils at the rate of 5.04 g kg^−1^ soil.

The soil is classified as a Hapli-Udic Cambisol (WRB). The initial soil properties of control soil were pH 7.10 (1:2.5, w/v), EC 0.13 ms cm^−1^ (1:5, w/v), available N 87.7 mg kg^−1^, available P 18.9 mg kg^−1^, available K 147.6 mg kg^−1^, total N 0.15 g kg^−1^, and total P 1.62 g kg^−1^. Initial soil properties of overfertilization soil were pH 7.08 (1:2.5, w/v), EC 0.14 ms cm^−1^ (1:5, w/v), available N 90.5 mg kg^−1^, available P 23.5 mg kg^−1^, available K 154 mg kg^−1^, total N 0.17 g kg^−1^, and total P 1.81 g kg^−1^.

### Greenhouse experiment

Soils were transported to the greenhouse (123° 24′ E, 41° 31′ N), which was located at Shenyang Agricultural University, Shenyang, Liaoning Province. Soils were sieved through a 2-cm screen to remove debris and stone and then mixed homogeneously with fertilizers according to the treatments. All treatments were arranged in a randomized complete block design, with 3 replicates per treatment.

The experiment was conducted from March 2020 to July 2020, and tomato plants (“Kaide Zunyue”) were planted in each treatment. Each tomato plant had a single branch after pruning, and there were three clusters of fruits on the branch and five fruits per cluster. The air temperature inside the experimental greenhouse ranged from 15 to 35°C. All tomato plants received natural light only. After one growing period of cultivation, 1, 2, and 12 years of soil were obtained.

### Soil sampling

In each treatment, soil samples were collected in July 2020 *via* a five-point sampling method, and each treatment was repeated three times. The roots and rocks were removed, and soil was passed through a 2-mm sieve. One part of the samples was air-dried to measure basal soil properties, one part was frozen in liquid N_2_ immediately and saved at −80°C for future DNA extraction, and the other fresh soil samples were kept in ice boxes to be transported to the lab and stored at 4°C for enzymes and NH${}_{4}^{+}$/NO${}_{3}^{-}$-N analyses.

### Yield and basal soil properties analysis

Tomatoes were harvested as fresh vegetables. Soil pH (soil:distilled water, 1:2.5) was determined using a Thunder Magnetic SJ-3F pH Meter (INESA, Shanghai, China). The soil EC (soil:distilled water, 1:5) value was determined using a Thunder Magnetic DDS-307 EC Meter (INESA, Shanghai, China). Soil NH${}_{4}^{+}$-N and NO${}_{3}^{-}$-N were extracted by 2 M KCl (5:1 *v*/*w*) and analyzed using the SAN^++^ Continuous Flow Analyzer (Skalar, Netherlands; Blakemore et al., 1987). Soil total C (TC) and total N (TN) contents were measured using an elemental analyzer (Elementar III, Germany). Total phosphorus (TP) was determined by the molybdenum-blue method (Nobile et al., 2020). Total potassium (TK) was measured using flame photometry after soil digestion with NaOH (Lu, 2000). Soil available phosphorus (AP) was determined in sodium bicarbonate extraction following colorimetric measurement (Olsen and Sommers, 1982). Available potassium (AK) was determined in the ammonium acetate extraction following the flame ionization photometry (Lu, 2000).

### Enzyme assays

Activities of β-1,4-glucosidase (βG, substrate: 4-MUB-β-D-glucopyranoside), leucine aminopeptidase (LAP, substrate: L-Leucine-7-AMC), and peroxidase and polyphenol oxidase (PER and PPO, substrate: L-DOPA) were measured using fluorogenic methods with 4-methylumbelliferyl (MUB), 7-amino-4-methylcoumarin (AMC), and L-dihydroxyphenylalanine (DOPA), respectively. Enzyme assays were performed following the protocol established by German et al. (2011). For each sample, soil slurry was prepared by adding 125 ml of 50 mM sodium acetate buffer (pH 5.0) to 1.5 g of fresh soil, then homogenizing for 1 min. The supernatant was continuously stirred using a magnetic stir plate, and 200 μl aliquots were dispensed into 96-well microplates and further used for the analyses. For hydrolytic enzymes (i.e., βG and LAP), the final concentrations of the substrates were 224.7 and 189.6 μM, and for oxidative enzymes (i.e., PER and PPO), it was 68.4 μM. The hydrolytic enzymes were incubated for 2.5 h, and oxidative enzymes were incubated for 24 h in the dark at 25°C. The quantity of fluorescence (hydrolytic enzymes) was read at 360 nm excitation and 460 nm emission; absorbance (oxidative enzymes) was read at 450 nm using a Microplate Reader (BioTek, Synergy2, United States).

### 16S, ITS rRNA Gene Amplification, and Sequencing

Soil DNA was extracted using the E.Z.N.A.^®^ Soil DNA Kit (Omega Bio-Tek, Norcross, GA, United States) according to the manufacturer's instructions. DNA concentration and purity were determined using a spectrophotometer, and DNA integrity was checked on a 1% agarose gel. These primers target the V3-V4 regions of bacterial 16S rRNA genes and ITS2 regions of fungal ITS rRNA genes (Ghannoum et al., 2010; Mori et al., 2013). The primers F338/R806 (F338, 5′-ACTCCTACGGGAGGCAGCAG-3′; R806, 5′-GGACTACHVGGGTWTCTAAT-3′) and ITS1/ITS2 (ITS1, 5′-CTTGGTCATTTAGAGGAAGTAA-3′; ITS2, 5′-GCTGCGTTCTTCATCGATGC-3′) were selected. The PCR amplification of the 16S rRNA gene was performed as follows: initial denaturation at 95°C for 3 min, then 27 cycles of denaturing at 95°C for 30 s, annealing at 55°C for 30 s and extension at 72°C for 45 s, and a single extension at 72°C for 10 min. The PCR amplification of the ITS rRNA gene was performed as follows: initial denaturation at 95°C for 3 min, then 35 cycles of denaturing at 95°C for 30 s, annealing at 55°C for 30 s and extension at 72°C for 45 s, and a single extension at 72°C for 10 min. The PCR products were purified using the AxyPrep DNA Gel Extraction Kit (Axygen Biosciences, Union City, CA, United States) according to the manufacturer's instructions and quantified by the real-time quantitative PCR. Purified amplicons were pooled in equimolar and paired-end sequenced on an Illumina MiSeq PE300 platform (Illumina, San Diego, United States) according to the standard protocols by Majorbio Bio-Pharm Technology Co. Ltd. (Shanghai, China). The raw reads were deposited into the NCBI Sequence Read Archive (SRA) database (Accession Number: PRJNA839183).

Sequencing reads were demultiplexed, quality-filtered using fastp version 0.20.0 (Chen et al., 2018), and merged by FLASH version 1.2.7 (Magoč and Salzberg, 2011). Poor-quality sequences shorter than 50 bp and with an average quality score of less than 20 were discarded, and reads containing ambiguous characters were also discarded. Operational taxonomic units (OTUs) with a 97% similarity cutoff (Stackebrandt and Goebel, 1994; Edgar, 2013) were clustered using UPARSE version 7.1, and chimeric sequences were identified and removed. The taxonomy of each OTU representative sequence was analyzed using RDP Classifier version 2.2 (Wang et al., 2007) against the 16S rRNA database (Rdp v11.5) and the ITS rRNA database (Unite v8.0), using a confidence threshold of 0.7.

### Statistical analyses

One-way ANOVA, mixed-effects ANOVA, and independent sample *T*-test were performed to reveal the significant differences in the crop yields and soil properties in the control and overfertilization treatment groups, respectively. Residuals of all ANOVA were checked for normality and homogeneity, and if assumptions were met, the Tukey's test was done and *p* < 0.05. When the interaction of fertilization and cropping duration is significant on soil properties, an independent sample *T*-test was used for cultivation durations in each fertilization treatment, and one-way ANOVA was used for cultivation durations in each fertilization treatment. When the interaction of fertilization and cropping duration is not significant on soil properties, one-way ANOVA was used for all treatments. All ANOVA analyses were conducted in SPSS 19.0 (IBM Corp., New York, United States). All experimental data are expressed as an average of three replicates with standard deviations.

Bacterial alpha-diversity (Shannon index and Chao1 index) was calculated with 10 times subsampling using Mothur software (version 1.30.2). Non-metric multidimensional scaling (NMDS) analyses based on Bray-Curtis dissimilarity matrices were performed to describe the structure of microbial community under treatments. Relationships between soil properties and microbial community composition were revealed by redundancy analysis (RDA). Variance inflation factors (VIFs) were used to detect multicollinearity, and factors with VIFs below 10 were retained. A multiple stepwise linear regression was used in SPSS 19.0 to select the most important contributors to explain the microbial communities. Residuals from the regression model were checked for normality as well.

Structural equation modeling (SEM) has been used to examine the direct or indirect effects of cropping duration, soil properties, and microbial community characteristics on crop yields. SEM analysis was performed using AMOS 24.0 (AMOS IBM, United States). The fitness of the model was evaluated *via* a non-significant chi-square test (*p* > 0.05), low χ^**2**^ (<7), high goodness-of-fit index (GFI > 0.95), and low root square mean errors of approximation (RMSEA < 0.08; Byrne and Erlbaums, 2009).

## Results

### Tomato yields and soil chemical properties

**Figure 1:**
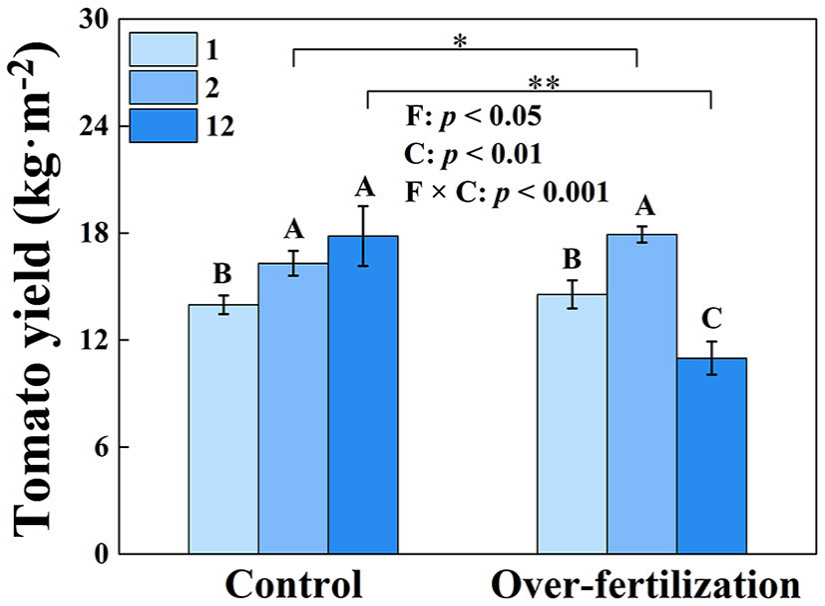
Effects of fertilization on tomato yield. Values are means ± SD (*n* = 3). * and ** indicate significant differences between fertilization under the same cropping duration at *p* < 0.05 and *p* < 0.01, respectively. Different letters (A, B, and C) above the bars indicate significant (*p* < 0.05) differences between cropping durations under the same fertilization treatments. F, fertilization; C, cropping duration.

Tomato yield significantly enhanced from 1- to 2-year-old soils in both control and overfertilization. However, overfertilization decreased tomato yield by 39% from 2 to 12 years. Under overfertilization, the yield decreased by 38% in the 12-year soil, compared to the control (Figure 1).

Soil pH was significantly reduced by 0.34 and 1.65 units from 1 to 12 years in control and overfertilization, respectively. Overfertilization showed a greater decrease in soil pH (by 4.85 times) than the control in 12-year soil (Table 2). Soil EC and concentrations of soil TC, TN, TP, AN, AP, AK, NH4^+^-N, and NO${}_{3}^{-}$-N enhanced with the increase of tomato cropping duration. Higher content of soil TC, TN, TP, AN, AP, AK, NH4^+^-N, and NO${}_{3}^{-}$-N were found under overfertilization compared to the control in the 12-year soil (Tables 1, 2).

**Table 1:** Mixed effect ANOVA for the effects of fertilization, crops, and their interaction on soil properties.

	**Fertilization**			**Cropping duration**			**Fertilization** × **Cropping duration**		
	**SS**	**F**	**Sig**.	**SS**	**F**	**Sig**.	**SS**	**F**	**Sig**.
Yield	10.9	21.7	**0.010**	30.8	14.4	**0.002**	64	30	**<0.001**
pH	1.3	135.6	**<0.001**	3.4	397.8	**<0.001**	1.5	182	**<0.001**
EC (ms·cm^−1^)	502,904	329.6	**<0.001**	740,030	491.8	**<0.001**	321,931	214	**<0.001**
TC (g·kg^−1^)	15.1	214.3	**<0.001**	94	212.4	**<0.001**	10.3	23.4	**<0.001**
TN (g·kg^−1^)	1.1	479.7	**<0.001**	1.5	128.4	**<0.001**	0.1	1.9	0.212
TP (g·kg^−1^)	256.2	3,433	**<0.001**	399	107.8	**<0.001**	0.6	0.2	0.851
TK (g·kg^−1^)	630.7	63.4	**<0.001**	527.5	39.2	**<0.001**	43.8	3.3	0.093
AN (mg·kg^−1^)	23,213	345.9	**<0.001**	9,294	160.2	**<0.001**	1,154	19.9	**0.001**
AP (mg·kg^−1^)	0.1	212.5	**<0.001**	0.1	158.4	**<0.001**	0.1	5.3	**0.035**
AK (mg·kg^−1^)	1.7	82.6	**<0.001**	1.3	43	**<0.001**	0.3	11.3	**0.005**
NH${}_{4}^{+}$-N (mg·kg^−1^)	258	64.3	**<0.001**	324.1	24.7	**<0.001**	285.5	21.7	**0.001**
NO${}_{3}^{-}$-N (mg·kg^−1^)	414.9	109.1	**<0.001**	1,187	65.5	**<0.001**	77.9	4.3	0.054
BG (nmol MUB g^−1^·soil·h^−1^)	29.4	6.9	0.058	560.2	91.5	**<0.001**	183	29.9	**<0.001**
LAP (nmol MUB g^−1^·soil·h^−1^)	3,744	511	**<0.001**	510.7	16.2	**0.002**	665.9	21.2	**0.001**
PER (nmol AMC g^−1^·soil·h^−1^)	0.2	1.1	0.345	12	5.2	**0.035**	14.7	6.4	**0.022**
PPO (nmol AMC g^−1^·soil·h^−1^)	0.3	0.2	0.676	35.2	36.7	**<0.001**	29.5	30.7	**<0.001**

EC, electrical conductivity; TC, total carbon; TN, total nitrogen; TP, total phosphorous; TK, total potassium; AN, available nitrogen; AP, available phosphorous; AK, available potassium; NH${}_{4}^{+}$-N, ammonium nitrogen; NO${}_{3}^{-}$-N, nitrate nitrogen; BG, β-1,4-glucosidase; LAP, leucine aminopeptidase; PER, peroxidase; PPO, polyphenol oxidase. The significance (sig.) was calculated based on sum of squares (SS). Sig. < 0.05 means significant, sig. > 0.05 means not significant.

**Table 2:** Effects of fertilization on soil characteristics under tomato cropping.

**Cropping duration**	**pH**		**EC (ms**·**cm**^**−1**^**)**		**TC (g**·**kg**^**−1**^**)**		**TN (g**·**kg**^**−1**^**)**		**TP (g**·**kg**^**−1**^**)**	
	**Control**	**OF**	**Control**	**OF**	**Control**	**OF**	**Control**	**OF**	**Control**	**OF**
1	7.07 ± 0.05A	7.02 ± 0.05A	0.14 ± 0.02C	0.21 ± 0.01C(**)	1.37 ± 0.13C	5.24 ± 0.11C(**)	0.17 ± 0.01e	0.76 ± 0.04c	1.79 ± 0.30e	8.83 ± 1.66c
2	6.97 ± 0.08A	6.77 ± 0.06B(*)	0.20 ± 0.02B	0.43 ± 0.03B(**)	6.29 ± 0.23B	6.53 ± 0.54B	0.53 ± 0.02d	1.03 ± 0.12b	4.70 ± 0.58d	12.39 ± 0.97b
12	6.73 ± 0.10B	5.37 ± 0.11C(**)	0.31 ± 0.02A	1.01 ± 0.06A(**)	8.20 ± 0.46A	9.59 ± 0.66A(*)	0.96 ± 0.07b	1.38 ± 0.08a	12.56 ± 0.27b	20.46 ± 1.84a
**Cropping duration**	**AP (g**·**kg**^−1^**)**		**AK (g**·**kg**^−1^**)**		**AN (g**·**kg**^−1^**)**		**NH**${}_{4}^{+}$**-N (mg**·**kg**^−1^**)**		**NO**${}_{3}^{-}$**-N (mg**·**kg**^−1^**)**	
	**Control**	**OF**	**Control**	**OF**	**Control**	**OF**	**Control**	**OF**	**Control**	**OF**
1	0.02 ± 0.01C	0.10 ± 0.01C(**)	0.20 ± 0.03B	0.44 ± 0.05B(**)	0.05 ± 0.00C	0.14 ± 0.01B(**)	2.07 ± 0.25A	3.78 ± 0.67B(*)	2.48 ± 0.14c	6.35 ± 1.71c
2	0.06 ± 0.01B	0.16 ± 0.00B(**)	0.53 ± 0.08A	1.19 ± 0.27A(*)	0.09 ± 0.01B	0.14 ± 0.01B(**)	2.20 ± 0.40A	4.37 ± 1.37B	6.62 ± 0.36c	17.92 ± 3.97b
12	0.09 ± 0.01A	0.21 ± 0.01A(**)	0.46 ± 0.09A	1.38 ± 0.11A(**)	0.11 ± 0.01A	0.18 ± 0.00A(***)	2.68 ± 0.25A	21.52 ± 5.63A(*)	17.35 ± 2.33b	30.98 ± 4.43a

Values are means of three replicates ± SD (n = 3). Different letters (a, b, c, d, and e) in the same edaphic factor indicate significant (p < 0.05) differences between all treatments. *, ** and *** indicate significant differences between different fertilization treatments under the same cropping duration at p < 0.05, p < 0.01 and p < 0.001, respectively. Different letters (A and B) in the same column indicate significant (p < 0.05) differences between cropping durations under the same fertilization treatments.

**Figure 2:**
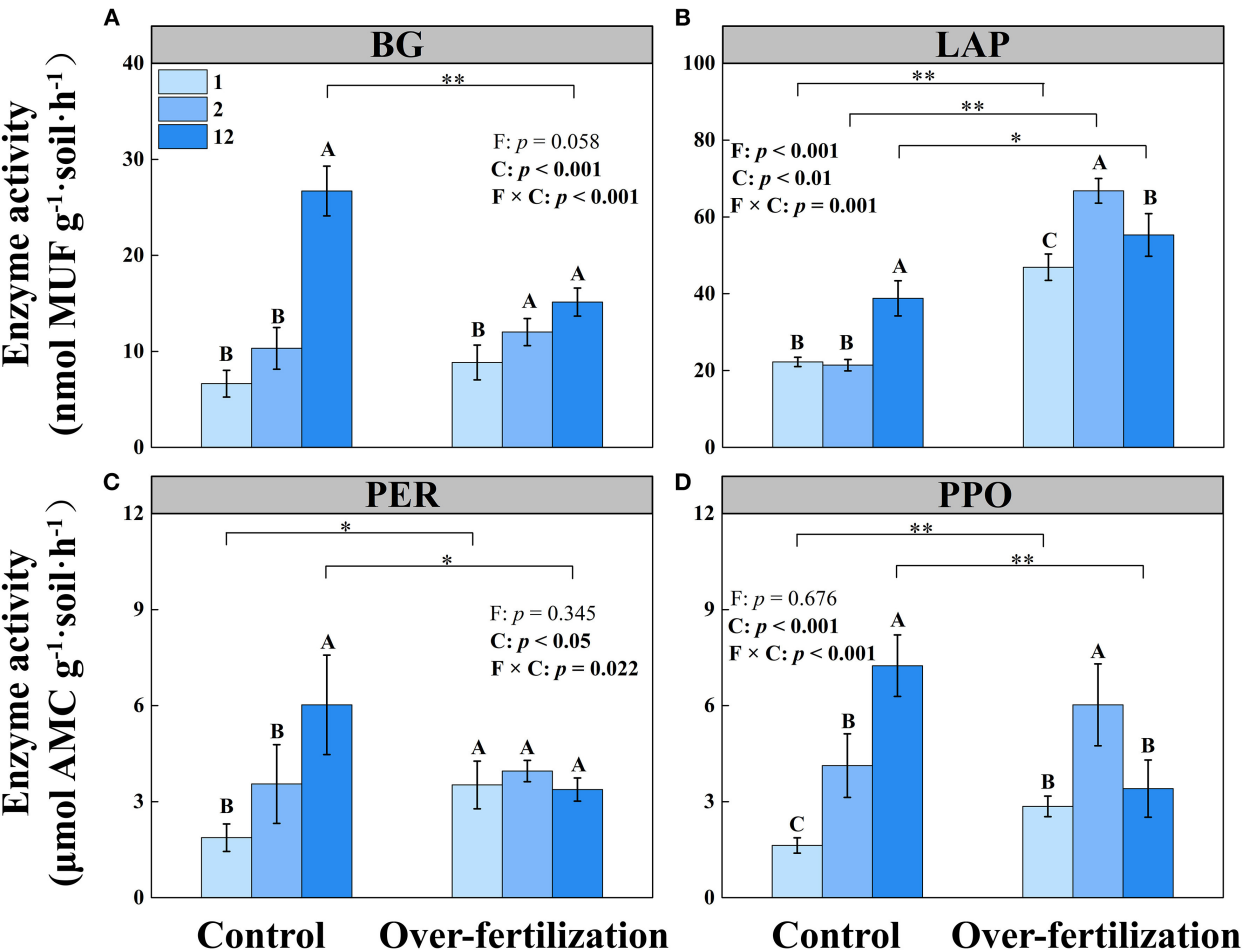
Effects of fertilization on soil enzyme activity under tomato cropping. **(A)** The activity of β-1,4-glucosidase (BG). **(B)** The activity of leucine aminopeptidase (LAP). **(C)** The activity of peroxidase (PER). **(D)** The activity of polyphenol oxidase (PPO). Values are means ± SD (*n* = 3). * and ** indicate significant differences between fertilization under the same cropping duration at *p* < 0.05 and *p* < 0.01, respectively. Different letters (A, B, and C) above the bars indicate significant (*p* < 0.05) differences between cropping durations under the same fertilization treatments. F, fertilization; C, cropping duration.

The activities of BG, LAP, PER, and PPO increased with the cropping duration under control, while the activity of PER stayed constant among different continuous cropping years under overfertilization. Overfertilization caused an increase in the activities of BG, LAP, and PPO from 1- to 2-year soils and a decrease in the activities of LAP and PPO from 2- to 12-year soils (Table 1 and Figure 2).

### Soil microbial alpha-diversity

A higher tomato cropping duration resulted in a decrease in the microbial diversity and richness of bacteria under control. Overfertilization enhanced the microbial diversity and richness of bacteria in 1- to 2-year soils and then decreased them in 2- to 12-year soils. Compared with control, overfertilization significantly decreased the bacterial diversity and richness under the same cropping duration.

**Figure 3:**
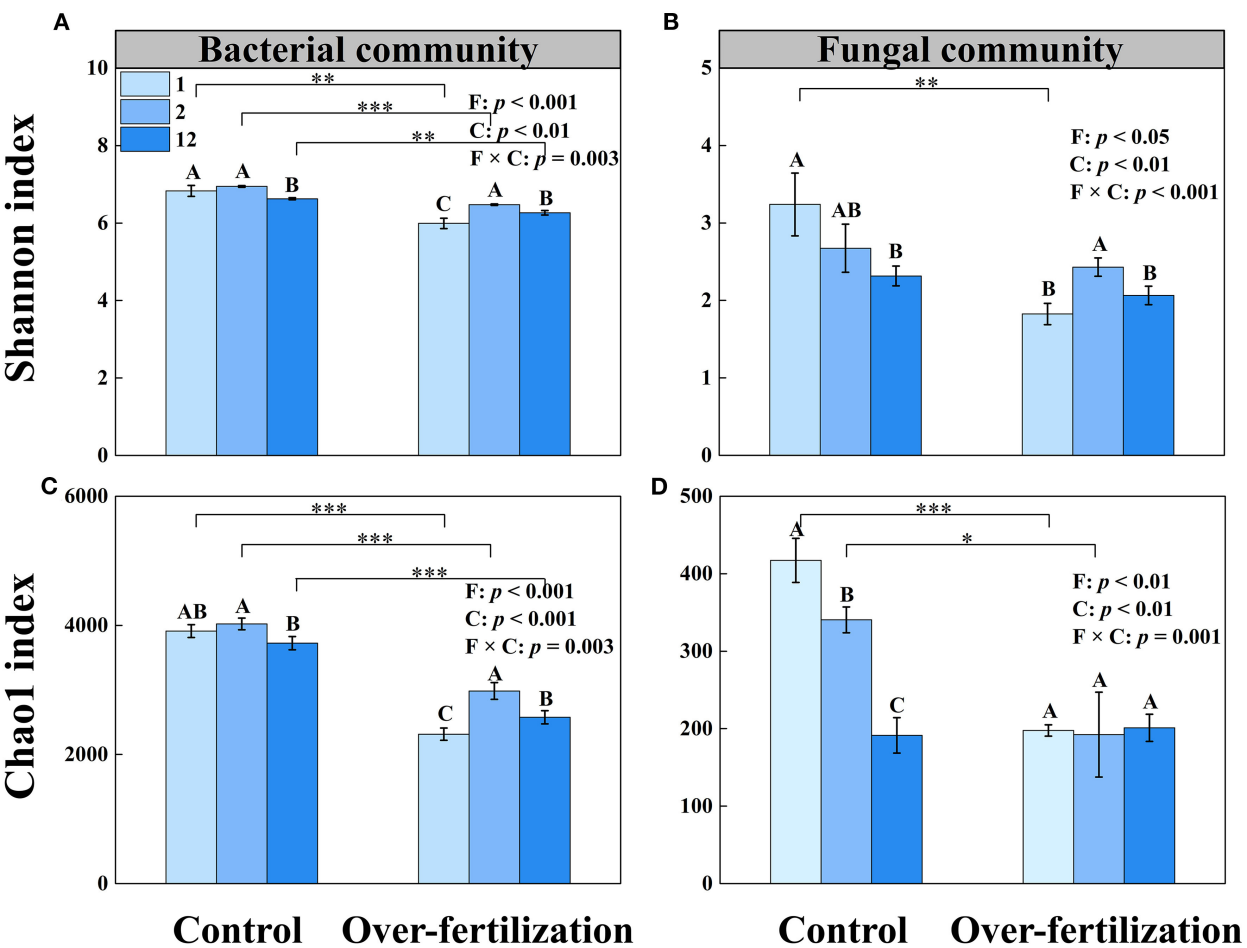
Effects of fertilization on soil microbial α-diversity under tomato cropping. **(A)** The variations of bacterial Shannon index. **(B)** The variations of fungal Shannon index. **(C)** The variations of bacterial Chao1 index. **(D)** The variations of fungal Chao1 index. Values are means ± SD (*n* = 3). *, **, and *** indicate significant differences between fertilization under the same cropping duration at *p* < 0.05, *p* < 0.01, and *p* < 0.001, respectively. Different letters (A, B, and C) above the bars indicate significant (*p* < 0.05) differences between cropping durations under the same fertilization treatments. F, fertilization; C, cropping duration.

The fungal diversity and richness decreased with the increase in tomato cropping duration for the control. Overfertilization resulted in an increase in fungal diversity from 1- to 2-year soils, followed by a decrease (Figure 3). Lower fungi diversity and richness were observed in the overfertilization than in the control (1- and 2-year soils).

### Soil microbial communities

**Figure 4:**
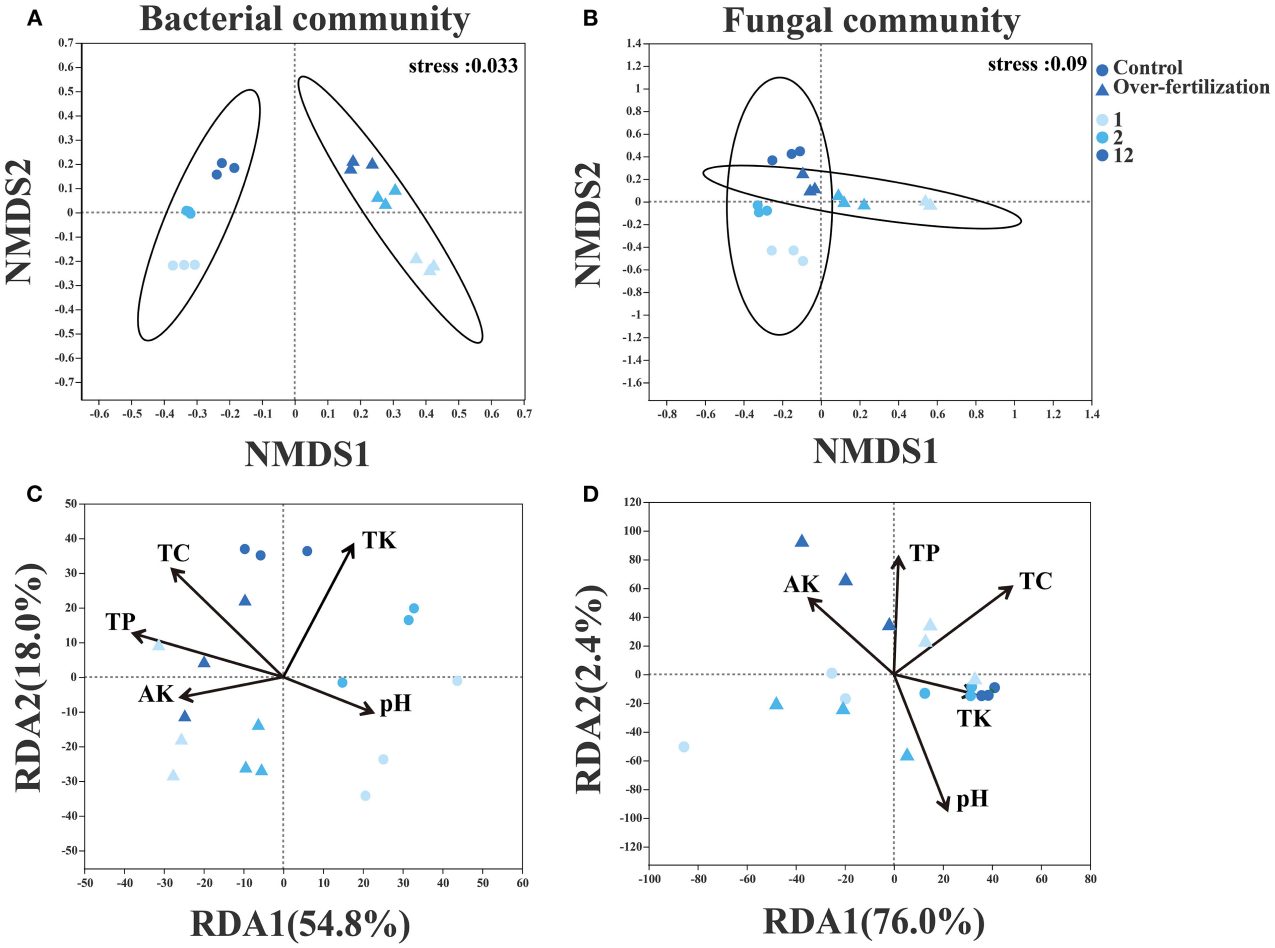
Effects of fertilization on microbial community composition and structure under tomato cropping. **(A)** The variations of bacterial NMDS under different treatments. **(B)** The variations of fungal NMDS under different treatments. **(C)** The variations of bacterial RDA under different treatments. **(D)** The variations of fungal RDA under different treatments. Variance inflation factors (VIFs) were used to detect multicollinearity, and factors with VIFs under 10 were retained. TC, total carbon; TP, total phosphorous; TK, total potassium; AK, available potassium.

For the bacterial community structure, a distinct separation was observed between control and overfertilization by NMDS axis1 (ANOSIM, *p* = 0.001). Fungal community structures in 1- and 2-year soils were separated between the control and overfertilization by NMDS axis1, and a distinct separation between various cropping duration was only observed under overfertilization by NMDS axis1 (ANOSIM, *p* = 0.002; Figures 4A,B).

Edaphic factors (i.e., pH, TC, TK, TP, and AK) explained 72.8% and 78.4% of the variation by the first two constrained axes of RDA in the community structure of bacteria and fungi, respectively. Soil TC, TK, and TP were positively correlated with the variations in the bacterial community. Soil pH, TP, and TC were positively correlated with the variations in the fungal community (Figures 4C,D).

### Soil-specific microbial taxa enriched

The multiple stepwise linear regression analysis showed that the bacterial community was influenced by *Gp6* and *Gemmatimonas* as they explained up to 83.9% (*p* < 0.01) of the variability. The soil fungal community was primarily explained by *Pseudaleuria* as it explained up to 74.0% (*p* < 0.01) of the variation (Table 3).

**Table 3:** Multiple stepwise linear regression analysis.

**Dependent variables**	**Explanatory variables**	**Coefficients**	**Stand error**	***t*-value**	**Significance level**	***R*^2^ (adjusted)**
Bacterial community	Constant	1.09	0.21	5.18	**<0.01**	
	*Gp6*	−0.04	0.10	−4.32	**<0.01**	0.71
	*Gp6* and *Gemmaatimonas*	−0.40	0.11	−3.72	**<0.01**	0.84
Fungal community	Constant	−0.12	0.04	−3.06	**<0.01**	
	*Pseudaleuria*	0.02	0.01	7.03	**<0.01**	0.74

The significance level < 0.05 means significant.

**Figure 5:**
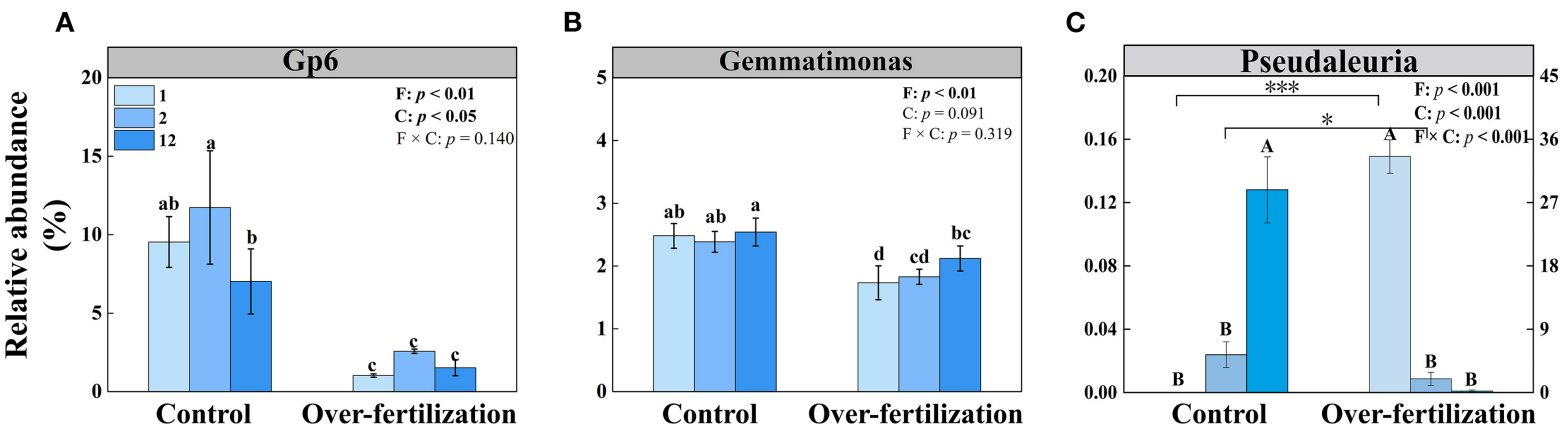
Effects of fertilization on the relative abundances of bacteria and fungi under tomato cropping. **(A)** The relative abundance of *Gp6*. **(B)** The relative abundance of *Gemmatimonas*. **(C)** The relative abundance of *Pseudaleuria*. Values are means ± SD (*n* = 3). Different letters (a, b, c, d, and e) above the bars indicate significant (*p* < 0.05) differences between all treatments. * and *** indicate significant differences between different fertilization treatments under the same cropping duration at *p* < 0.05 and *p* < 0.001, respectively. Different letters (A and B) above the bars indicate significant (*p* < 0.05) differences between cropping durations under the same fertilization treatments. F, fertilization; C, cropping duration.

The relative abundance of Gp6 was constant for the soils having various cultivation ages in both control and overfertilization. The relative abundance of *Gp6* was lower under overfertilization compared to the control (Figure 5A). The relative abundance of *Gemmatimonas* was the same in control, while overfertilization enhanced it with the increase in tomato cropping duration. The relative abundance of *Gemmatimonas* was lower under overfertilization compared to the control (Figure 5B). Relative abundance of *Pseudaleuria* was reduced under overfertilization, while control enhanced it with the increase in tomato cropping duration. Overfertilization led to a higher relative abundance of *Pseudaleuria* than control in 1- and 2-year soils (Figure 5C), but no difference was observed in 12-year soil (Figure 5C).

### Relationship between soil properties and crop yields

**Figure 6:**
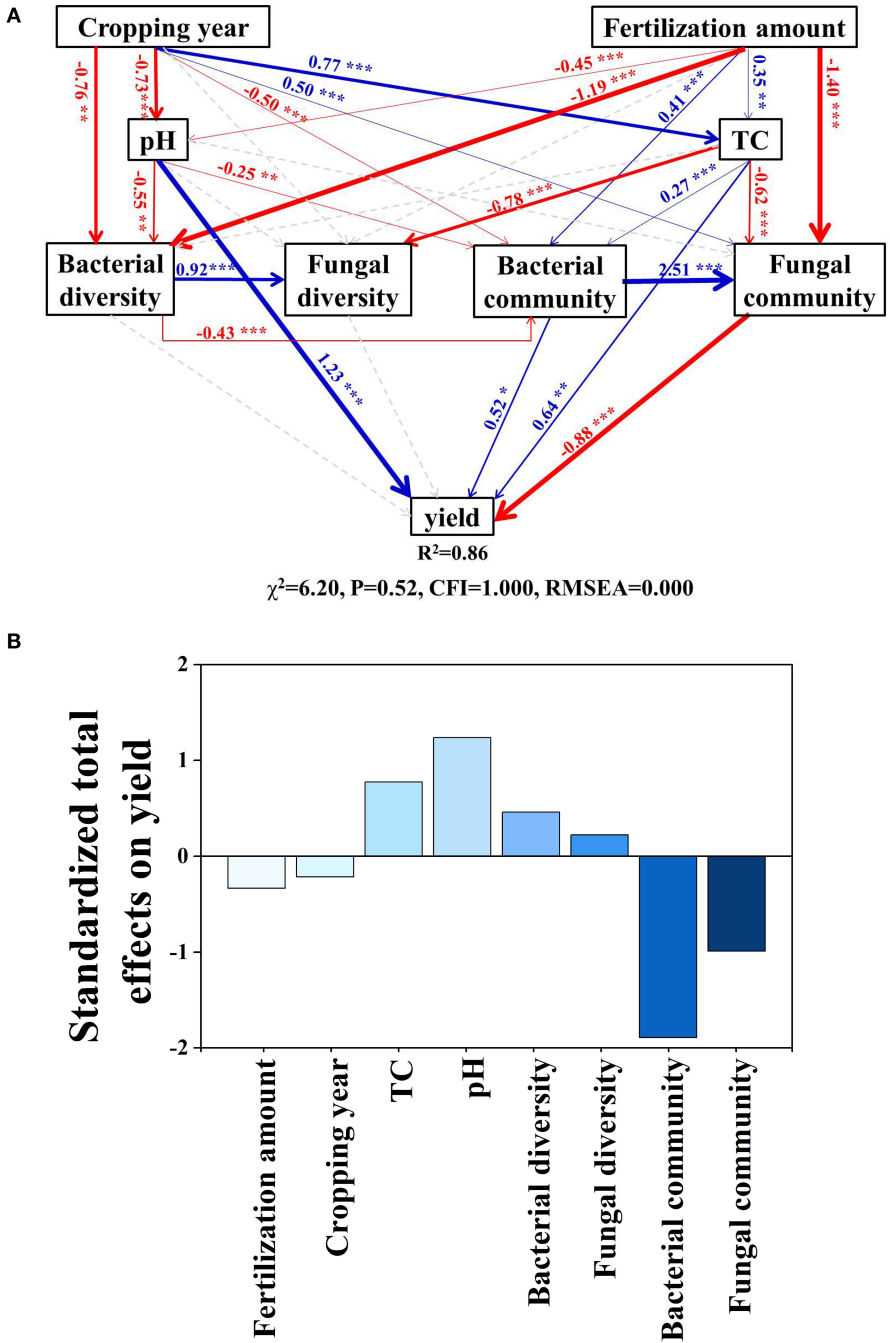
Effects of multiple factors on crop yield. **(A)** Structural equation modeling based on the effects of cropping durations, fertilization, soil properties (pH and TC), bacterial diversity and community composition, fungal diversity and community composition on crop yield. **(B)** Standardized total effects. Red arrows indicate significant negative and blue positive relationships. Dashed arrows represent non-significant relationships. Arrow width indicates the strength of the relationships. Numbers at arrows are standardized path coefficients. *R*^2^ denotes the proportion of variance explained by the model. *p < 0.05; **p < 0.01; ***p < 0.001. TC, total carbon.

The SEM model explained 86% of the variation in yield (Figure 6A). Cropping duration had direct positive effects on soil TC and the fungal community but had direct negative effects on soil pH, bacterial diversity, and community structure. These pathways led to a negative total effect of cropping duration on tomato yield (effect size = −0.23, Supplementary Table 1-1). Fertilization amount had direct positive effects on soil TC and bacterial community but had direct negative effects on soil pH, bacterial diversity, and the fungal community. Fertilization amount had a negative total effect on tomato yield (effect size = −0.33, Supplementary Table 1-1). Standardized total effects showed that the change in tomato yield was mainly driven by the bacterial community, followed by soil pH, fungal community, soil TC, bacterial diversity, fertilization amount, fungal diversity, and cropping year (Figure 6B).

## Discussion

### Overfertilization deteriorated the tomato yield

Fertilization generated a greater effect on tomato yield than cropping duration (Figure 1). A decrease in tomato yield was observed for overfertilization in 12-year soil. Overfertilization can alter soil properties and influence tomato shoot and root growth (Omay et al., 1997; Sainju et al., 2000). When the N application rate was increased from 375 kg N ha^−1^ to 1,123 kg ha^−1^, the nitrogen use efficiency was significantly reduced (Liu et al., 2004). Over NO_3_–N accumulation resulting from long-term fertilizer input (Table 1) increased N leaching and N_2_O, and NH_3_ emissions, which may cause water and air pollution, resulting in high N loss risk and causing a negative effect on the growth of tomatoes (Min et al., 2011; Wang and Xing, 2016; Yao et al., 2019). Furthermore, long-term overfertilization can drive soil acidification (Table 1), which degrades soil and environmental quality (Guo et al., 2010). Soil acidification promoted soil nitrification (Shang et al., 2014) and inhibited root growth (Wang et al., 2017). Long-term tomato monoculture with intensive inorganic fertilizer addition enhanced soil salinity (shown as a high EC value; Zhao et al., 2019), which reduced the availability of plant water uptake by decreasing the osmotic potential of the soil solution, causing disruption of nutritional balance, which eventually reduced tomato yield (Kahlaoui et al., 2011). In addition, overfertilization suppressed the production of BG, PER, and PPO (Figure 2) because enzyme activity is predominantly controlled by temperature and pH, which affect enzyme kinetics through changes in substrate binding and stability (Puissant et al., 2019). Thus, overfertilization has a negative effect on tomato yield under long-term continuous cropping.

### The response of microbial communities to overfertilization under long-term tomato cropping

Overuse of inorganic fertilization reduced the microbial diversity and richness and changed the microbial community composition (Figure 3). The bacterial community and the fungal community were influenced by variations of *Gp6, Gemmatimonas*, and *Pseudaleuria*, respectively (Table 3). The relatively high abundances of *Gp4, Gp6*, and *Gp16*, which belong to *Acidobacteria*, reflected the neutral pH of the soil (Jones et al., 2009; Zheng et al., 2019). The relative abundance of *Gp6* tends to be more enriched in mildly acidic soil, which is found in control. Overfertilization decreased soil pH and reduced the relative abundance of *Gp6* because N enrichment inhibited the growth of oligotrophic bacteria (Dai et al., 2018). Control induced a high relative abundance of *Gemmatimonas*, which contributes to the degradation of cellulose (Wang et al., 2017). Members of the *Gemmatimonas* genus are known to contribute to soil organic carbon sequestration (Guo et al., 2016), and it can be against plant pathogens and improvement of soil nutrients (Liu et al., 2020). Furthermore, *Gemmatimonas* is related to the metabolism and transformation of nitrogen, which showed strong positive associations with total nitrogen and reduced the potent greenhouse gas N_2_O under both aerobic and anaerobic conditions (Li et al., 2017; Park et al., 2017). Some bacterial strains belonging to the genus *Gemmatimonas* could perform bacteriochlorophyll-based chlorophototrophy, which possesses an expanded gene repository for coping with oxidative stresses (Zeng et al., 2021). In addition, a high abundance of *Gemmatimonas* was found in the healthy wheat plant rhizosphere, which indicated that *Gemmatimonas* can help suppress diseases and promote plant growth (Yin et al., 2013; Li et al., 2017). A combination of organic and inorganic fertilizers is beneficial for the relative abundance of certain favorable fungal taxa such as *Pseudaleuria*, which inhibits crop pathogens (Ding et al., 2017; Xiang et al., 2020). *Pseudaleuria* was the most abundant in healthy soils, which was involved in the interactions with plant roots to suppressive soil diseases (Xu et al., 2012). Relative abundance of *Pseudaleuria* increased under overfertilization in the short term but reduced markedly in the long term, while control showed the contrast effects. Thus, soil beneficial microorganisms decrease when the greenhouse ecosystem is exposed to consistent overfertilization.

### Linkage of soil microbial community composition and tomato yield in response to fertilization

Based on SEM model analyses, the decrease in tomato yield caused by overfertilization was mainly due to the direct effect of pH and the indirect effects of bacterial community composition (Figure 6 and Supplementary Table 1). Continuous cropping and fertilization not only directly but also indirectly affected soil microorganisms through soil pH (Ning et al., 2020), salt (Zhou et al., 2016), nitrogen content (Liu et al., 2021), and availability of organic compounds (Ning et al., 2021). The pH played a more important role in the microbial diversity, while TC played a more important role in the microbial community structure (Supplementary Table 1), especially the bacterial one because fungi have wider enzymatic capabilities and a higher capacity for the decomposition of plant polymers than bacteria do, which means that fungi provide bacteria with resources that the bacteria are not able to acquire on their own (Romani et al., 2006; Sun et al., 2018).

The microbial community played an important role in tomato yield (Figure 6). There are several reasons for this: (i) continuous high fertilizer loading increased N availability (Table 2), which reduces the belowground allocation of recent photosynthate C by plants (Högberg et al., 2010), and as a consequence, the microbial biomass C and basal respiration rates (Ramirez et al., 2012), and the abundance of community members decline, such as ectomycorrhizal fungi (Choma et al., 2017); (ii) fertilization intensities can alter microbial community structure (Figures 3, 4), and *Olpidium* (a potential phytopathogenic), which caused the negative effect on tomato plants (Usero et al., 2021), was positively affected by inorganic fertilizer; (iii) the relative abundance of *Actinobacteria* was decreased under long-term overfertilization, which inhibited the direct antagonism between *Actinobacteria* and antibiotics produced by fungi pathogens (Zhao et al., 2019). The number and types of beneficial microorganisms decreased, and harmful microorganisms enhanced in the soil unbalanced the microbial community, which can cause a significant decrease in soil quality and crop growth (Li et al., 2019; Zhou et al., 2021). Thus, the decrease in tomato yield under overfertilization in our study has a close relationship with the altering of soil pH, soil TC, and microbial community composition.

## Conclusion

Long-term overfertilization enhanced soil EC, increased nutrient enrichment, and decreased soil pH. In contrast, the control had a lower soil EC and soil nutrient concentrations and had a higher soil pH than overfertilization. Overfertilization significantly decreased bacterial diversity and richness than control in 12-year soils. The relative abundance of *Gp6* and *Gemmatimonas* was significantly decreased in overfertilization compared to the control, which destroyed the balance of the microbial community. Furthermore, overfertilization decreased the tomato yield by 38% in 12 years compared to the control. The results confirmed that overfertilization has a negative effect on tomato yield under long-term continuous cropping. Collectively, long-term overfertilization intensified soil acidification and altered microbial community composition, thus decreasing tomato yield. Therefore, appropriate reductions in the use of inorganic fertilizers in greenhouse cultivation systems are critical to soil sustainability.

## Data availability statement

The datasets presented in this study can be found in online repositories. The names of the repository/repositories and accession number(s) can be found in the article/Supplementary material.

## Author contributions

TL, HF, ZS, and QiaS designed the experiment. QiaS, XS, ZW, and QinS conducted the experiment and collected data for preliminary analysis. QiaS and HF further analyzed the data and prepared the manuscript. All authors contributed to the article and approved the submitted version.

## Funding

This study was supported by the National Natural Science Foundation of China (Grant No. 31902093) and the National Key R&D Program of China (Grant No. 2019YFD1001900).

## Conflict of interest

The authors declare that the research was conducted in the absence of any commercial or financial relationships that could be construed as a potential conflict of interest.

## Publisher's note

All claims expressed in this article are solely those of the authors and do not necessarily represent those of their affiliated organizations, or those of the publisher, the editors and the reviewers. Any product that may be evaluated in this article, or claim that may be made by its manufacturer, is not guaranteed or endorsed by the publisher.

## Abbreviations

AK: Available potassium
AMC: 7-amino-4-methylcoumarin
AN: Available nitrogen
AP: Available phosphorous
BG, β-1: 4-glucosidase
C: Cropping duration
DOPA: L-dihydroxyphenylalanine
EC: Electrical conductivity
F: Fertilization
LAP: Leucine aminopeptidase
MUB: 4-methylumbelliferyl
NH${}_{4}^{+}$-N: Ammonium nitrogen
NMDS: Non-metric multidimensional scaling
NO${}_{3}^{-}$-N: Nitrate nitrogen
OF: Overfertilization
PER: Peroxidase
PPO: Polyphenol oxidase
RDA: Redundancy analysis
SEM: Structural equation modeling
SRA: Sequence read archive
TC: Total carbon
TK: Total potassium
TN: Total nitrogen
TP: Total phosphorous
VIFs: Variance inflation factors.
