# Supplementary material for: Overfertilization reduces tomato yield under long-term continuous cropping system via regulation of soil microbial community composition
Source: Front Microbiol. 2022 Aug 4;13:952021. doi: 10.3389/fmicb.2022.952021 (PMC9386239; doi:10.3389/fmicb.2022.952021)
Supplement: Supplementary file 1 [file Table_1.docx]

Supplementary Material

# Supplementary Tables

**Table 1-1** Standardized total effects of structural equation models

|  | **Fertilization**  **amount** | **Cropping**  **duration** | **TC** | **pH** | **Bacterial**  **diversity** | **Fungal**  **diversity** | **Bacterial**  **community** | **Fungal**  **community** |
| --- | --- | --- | --- | --- | --- | --- | --- | --- |
| **TC** | 0.353 | 0.770 | 0.000 | 0.000 | 0.000 | 0.000 | 0.000 | 0.000 |
| **pH** | -0.454 | -0.730 | 0.000 | 0.000 | 0.000 | 0.000 | 0.000 | 0.000 |
| **Bacterial diversity** | -0.835 | -0.132 | 0.303 | -0.546 | 0.000 | 0.000 | 0.000 | 0.000 |
| **Bacterial community** | 0.968 | -0.057 | 0.145 | -0.013 | -0.426 | 0.000 | 0.000 | 0.000 |
| **Fungal diversity** | -0.653 | -0.338 | -0.497 | -0.653 | 0.918 | 0.000 | 0.000 | 0.000 |
| **Fungal community** | 0.741 | -0.237 | -0.254 | 0.127 | -1.068 | 0.000 | 2.508 | 0.000 |
| **Yield** | -0.334 | -0.228 | 0.811 | 1.181 | 0.557 | 0.101 | -1.684 | -0.880 |

**Table 1-2** Standardized direct effects of structural equation models

|  | **Fertilization**  **amount** | **Cropping**  **duration** | **TC** | **pH** | **Bacterial**  **diversity** | **Fungal**  **diversity** | **Bacterial**  **community** | **Fungal**  **community** |
| --- | --- | --- | --- | --- | --- | --- | --- | --- |
| **TC** | 0.353 | 0.770 | 0.000 | 0.000 | 0.000 | 0.000 | 0.000 | 0.000 |
| **pH** | -0.454 | -0.730 | 0.000 | 0.000 | 0.000 | 0.000 | 0.000 | 0.000 |
| **Bacterial diversity** | -1.190 | -0.764 | 0.303 | -0.546 | 0.000 | 0.000 | 0.000 | 0.000 |
| **Bacterial community** | 0.405 | -0.503 | 0.273 | -0.246 | -0.426 | 0.000 | 0.000 | 0.000 |
| **Fungal diversity** | 0.318 | 0.269 | -0.775 | -0.152 | 0.918 | 0.000 | 0.000 | 0.000 |
| **Fungal community** | -1.398 | 0.498 | -0.617 | 0.160 | 0.000 | 0.000 | 2.508 | 0.000 |
| **Yield** | 0.000 | 0.000 | 0.638 | 1.228 | -0.252 | 0.101 | 0.523 | -0.880 |

**Table 1-3** Standardized indirect effects of structural equation models

|  | **Fertilization**  **amount** | **Cropping**  **duration** | **TC** | **pH** | **Bacterial**  **diversity** | **Fungal**  **diversity** | **Bacterial**  **community** | **Fungal**  **community** |
| --- | --- | --- | --- | --- | --- | --- | --- | --- |
| **TC** | 0.000 | 0.000 | 0.000 | 0.000 | 0.000 | 0.000 | 0.000 | 0.000 |
| **pH** | 0.000 | 0.000 | 0.000 | 0.000 | 0.000 | 0.000 | 0.000 | 0.000 |
| **Bacterial diversity** | 0.355 | 0.632 | 0.000 | 0.000 | 0.000 | 0.000 | 0.000 | 0.000 |
| **Bacterial community** | 0.563 | 0.446 | -0.129 | 0.233 | 0.000 | 0.000 | 0.000 | 0.000 |
| **Fungal diversity** | -0.971 | -0.607 | 0.278 | -0.502 | 0.000 | 0.000 | 0.000 | 0.000 |
| **Fungal community** | 2.139 | -0.735 | 0.363 | -0.032 | -1.068 | 0.000 | 0.000 | 0.000 |
| **Yield** | -0.334 | -0.228 | 0.172 | -0.047 | 0.809 | 0.000 | -2.206 | 0.000 |
